# TBAJ-876, a 3,5-Dialkoxypyridine Analogue of Bedaquiline, Is Active against Mycobacterium abscessus

**DOI:** 10.1128/AAC.02404-19

**Published:** 2020-03-24

**Authors:** Jickky Palmae Sarathy, Uday S. Ganapathy, Matthew D. Zimmerman, Véronique Dartois, Martin Gengenbacher, Thomas Dick

**Affiliations:** a Department of Medicine, Yong Loo Lin School of Medicine, National University of Singapore, Singapore; b Center for Discovery and Innovation, Hackensack Meridian Health, Nutley, New Jersey, USA; c Department of Medical Sciences, Hackensack Meridian School of Medicine, Seton Hall University, Nutley, New Jersey, USA

**Keywords:** TBAJ-876, bedaquiline, *Mycobacterium abscessus*, NTM, nontuberculous mycobacteria

## Abstract

Lung disease caused by Mycobacterium abscessus is very difficult to cure, and treatment failure rates are high. The antituberculosis drug bedaquiline (BDQ) is used as salvage therapy against this dreadful disease. However, BDQ is highly lipophilic, displays a long terminal half-life, and presents a cardiotoxicity liability associated with QT interval prolongation. Recent medicinal chemistry campaigns resulted in the discovery of 3,5-dialkoxypyridine analogues of BDQ which are less lipophilic, have higher clearance, and display lower cardiotoxic potential. TBAJ-876, a clinical development candidate of this series, shows attractive *in vitro* antitubercular activity and efficacy in a murine tuberculosis model. Here, we asked whether TBAJ-876 is active against M. abscessus. TBAJ-876 displayed submicromolar *in vitro* activity against reference strains representing the three subspecies of M. abscessus and against a collection of clinical isolates. Drug-drug potency interaction studies with commonly used anti-M. abscessus antibiotics showed no antagonistic effects, suggesting that TBAJ-876 could be coadministered with currently used drugs. Efficacy studies, employing a mouse model of M. abscessus infection, demonstrated potent activity *in vivo*. In summary, we demonstrate that TBAJ-876 shows attractive *in vitro* and *in vivo* activities against M. abscessus, similar to its BDQ parent. This suggests that next-generation BDQ, with improved tolerability and pharmacological profiles, may be useful for the treatment of M. abscessus lung disease in addition to the treatment of tuberculosis.

## Abstract (precis)

Lung disease caused by Mycobacterium abscessus is very difficult to cure, and treatment failure rates are high. The antituberculosis drug bedaquiline (BDQ) is used as salvage therapy against this dreadful disease. However, BDQ is highly lipophilic, displays a long terminal half-life, and presents a cardiotoxicity liability associated with QT interval prolongation. Recent medicinal chemistry campaigns resulted in the discovery of 3,5-dialkoxypyridine analogues of BDQ which are less lipophilic, have higher clearance, and display lower cardiotoxic potential.

## INTRODUCTION

Nontuberculous mycobacteria (NTM) are environmental mycobacteria that can cause pulmonary disease (NTM-PD). The disease occurs mostly in patients with immunodeficiencies or preexisting pulmonary conditions such as cystic fibrosis or bronchiectasis (1–4). Infection typically occurs through contact with contaminated household or municipal water, which are habitats for NTM (5, 6). A recent study proposed that human-to-human transmission may also occur (7). NTM-PD is an emerging global health concern given that the number of cases is increasing worldwide (8, 9). The disease is prevalent in the United States, the Middle East, and Asia (10–13). Mycobacterium abscessus is one of the common NTM species identified in patients with NTM-PD (10, 13). M. abscessus presents as a complex of three closely related subtaxa, M. abscessus subsp. *abscessus*, M. abscessus subsp. *bolletii*, and M. abscessus subsp. *massiliense* (14).

Treatment of M. abscessus infections is largely based on recommendations derived from retrospective studies (15). The regimen typically consists of a macrolide (e.g., clarithromycin) combined with an aminoglycoside (amikacin) and a β-lactam (imipenem or cefoxitin) (1, 16, 17). Chemotherapy can be long-lasting, as negative sputum cultures for 1 year are recommended before cure can be declared (1, 17). Despite such stringent clinical practices, success rates are reported to be only 25 to 58%, disease often reoccurs (18–23), and adverse side effects such as gastrointestinal distress, ototoxicity, and nephrotoxicity are commonly experienced by patients (23). In addition, there is a shortage of alternative treatments when regimens fail, as M. abscessus is intrinsically resistant to many antibiotics (16, 24, 25). Treatment failure is often associated with inducible clarithromycin resistance (25), which is mediated by the *erm*(41) gene. Functional *erm*(41) can be present in M. abscessus subsp. *abscessus* and subsp. *bolletii* but is typically absent in M. abscessus subsp. *massiliense* (26, 27). Collectively, the issues associated with M. abscessus treatment highlight the need to develop new drugs or reposition existing antibiotics (28, 29).

**FIG 1:**
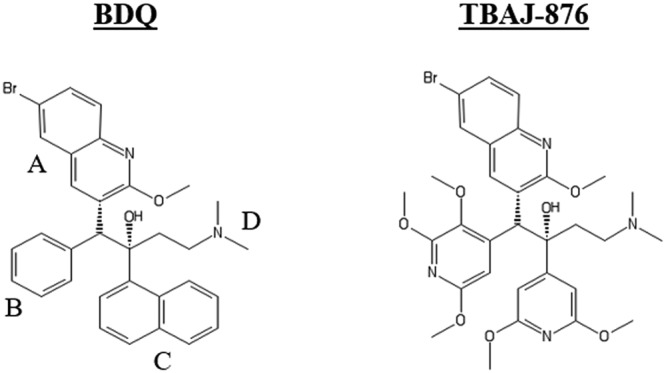
Structures of BDQ and TBAJ-876. TBAJ-876 was described as “compound 46” previously (45). The compound retains BDQ’s quinoline (A) and dimethylamino (D) groups. BDQ’s phenyl (B) and naphthalene (C) groups are replaced by the 2,3,5-trimethoxypyridin-4-yl and 3,5-dimethoxypyridin-4-yl groups, respectively.

Bedaquiline (BDQ) (Sirturo) (Fig. 1) is a first-in-class diarylquinoline (DARQ) (30) used for the treatment of multidrug-resistant tuberculosis (31, 32). This highly potent antituberculosis drug (30) exerts its antibacterial activity by inhibiting Mycobacterium tuberculosis F-ATP synthase (30, 33). Interestingly, BDQ is active against the M. abscessus complex (34–37) and has been utilized as salvage therapy for patients with persistent M. abscessus infection (38). However, BDQ has several pharmacological and toxicological liabilities. The drug is highly lipophilic (cLogP [logarithm of the drug’s partition coefficient between *n*-octanol and water] = 7.25), has a long terminal half-life, and accumulates in tissues (39, 40). Furthermore, the drug causes prolongation of the QT interval due to inhibition of cardiac human ether-a-go-go-related gene (hERG) potassium ion channels (39, 40). A recent medicinal chemistry campaign, conducted to address these issues, led to the discovery of 3,5-dialkoxypyridine analogues of BDQ (41–46). TBAJ-876 (Fig. 1) is a clinical development candidate that emerged from this series. It displays lower inhibition of hERG channels and higher clearance and retains *in vivo* efficacy against M. tuberculosis (45). We have shown that TBAJ-876 is bactericidal and as potent as BDQ against M. tuberculosis and retains BDQ’s on-target activity via binding to the c and ε subunits of the mycobacterial F-ATP synthase (47, 48).

TBAJ-876 is active not only against M. tuberculosis but also against the fast-growing saprophytic mycobacterial model organism Mycobacterium smegmatis (47), suggesting that the compound may display broad-spectrum antimycobacterial activity. The goal of the present work was to evaluate the clinical potential of TBAJ-876 against M. abscessus lung disease. *In vitro* activities of TBAJ-876 against reference strains and clinical isolates of the M. abscessus complex were measured, and the *in vivo* efficacy of this new compound was determined in a mouse model of M. abscessus infection.

## RESULTS

### TBAJ-876 is active against reference strains of the M. abscessus complex.

To determine whether TBAJ-876 is active against the three subspecies that form the M. abscessus complex, each subspecies reference strain was tested for its susceptibility to the compound in complete Middlebrook 7H9 broth. TBAJ-876 displayed high potency, with MICs ranging from 0.42 to 0.53 μM across all subspecies, within 2-fold of BDQ’s MIC (Table 1). These results suggest that TBAJ-876 is active against the M. abscessus complex, displaying a potency similar to that of the parent drug against all subspecies.

**TABLE 1:** Growth-inhibitory potency of TBAJ-876 against reference strains representing the subspecies of the M. abscessus complex in 7H9 and CAMH media^*a*^

DARQ	Type of medium	MIC against strain (μM)		
		M. abscessus subsp. *abscessus* ATCC 19977	M. abscessus subsp. *bolletii* CCUG 50184-T	M. abscessus subsp. *massiliense* CCUG 48898-T
TBAJ-876	7H9	0.48	0.53	0.42
	CAMH	1.05	0.40	1.10

BDQ	7H9	0.56	0.76	0.64
	CAMH	1.20	0.50	0.84

a The experiment was carried out three times independently, and the MICs are displayed as mean values. BDQ was used as a positive control. DARQ, diarylquinoline.

Since drug susceptibility testing for M. abscessus is often carried out in cation-adjusted Mueller-Hinton (CAMH) medium (49, 50), the potency of TBAJ-876 and BDQ was measured in this medium, revealing a minor increase in the MICs against M. abscessus subsp. *abscessus* ATCC 19977 and M. abscessus subsp. *massiliense* CCUG 48898-T for both compounds (Table 1). Thus, TBAJ-876 and BDQ exert similar potencies against reference strains of the M. abscessus complex in Middlebrook 7H9 and CAMH media.

### TBAJ-876 is active against clinical isolates of the M. abscessus complex.

Next, we determined whether this potent activity holds true against a panel of clinical isolates. This collection included M. abscessus Bamboo, an isolate previously sequenced (51) and used in drug screening campaigns by our group (52–54). TBAJ-876 was uniformly potent against M. abscessus Bamboo and other clinical isolates, with MICs ranging from 0.14 to 0.45 μM (Table 2), similar to the MICs observed for the subspecies reference strains (Table 1). Thus, TBAJ-876 is active *in vitro* against clinical isolates of the M. abscessus complex, including those resistant to clarithromycin, the pillar of M. abscessus treatment.

**TABLE 2:** Growth-inhibitory potency of TBAJ-876 against clinical isolates of the M. abscessus complex^*a*^

Isolate^*b*^	M. abscessus subsp.	*erm*(41) sequevar^*c*^	Clarithromycin susceptibility^*c*^	MIC (μM)	
				TBAJ-876	BDQ
Bamboo	*abscessus*	C28	Sensitive	0.46	0.55
M9	*abscessus*	T28	Resistant	0.30	0.36
M111	*massiliense*	Deletion	Sensitive	0.30	0.31
M199	*abscessus*	T28	Resistant	0.43	0.55
M232	*bolletii*	T28	Resistant	0.45	0.56
M337	*abscessus*	T28	Resistant	0.31	0.44
M421	*abscessus*	T28	Resistant	0.14	0.15
M422	*abscessus*	T28	Resistant	0.30	0.28
M506	*bolletii*	C28	Sensitive	0.30	0.42

a The experiment was carried out three times independently, and the MICs are displayed as mean values. BDQ was used as a positive control.

b M. abscessus Bamboo is a clinical isolate whose genome characterization was described previously (51). The other clinical isolates were identified to the species level and characterized as described previously (52).

c *erm*(41) is the methylase gene responsible for inducible clarithromycin resistance. The “C28” and “deletion” sequevars confer susceptibility to clarithromycin, while the “T28” sequevar confers inducible resistance against clarithromycin (26, 27).

### TBAJ-876 is bacteriostatic against M. abscessus *in vitro*.

**FIG 2:**
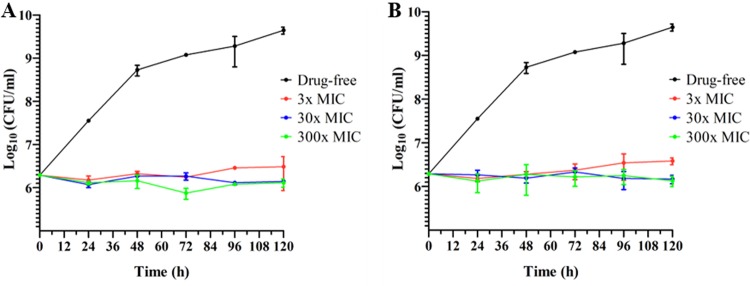
Bacteriostatic activity of TBAJ-876 against M. abscessus Bamboo *in vitro*. Shown is the growth and survival of M. abscessus Bamboo treated with 3-, 30-, and 300-fold the MIC of TBAJ-876 (A) or the positive-control BDQ (B) over a period of 5 days. The MICs of TBAJ-876 and BDQ are 0.46 μM and 0.55 μM, respectively (Table 2). The experiment was carried out three times independently, and the results are represented as mean values, with the standard deviations displayed as error bars. The graphs were generated using GraphPad Prism 5 software.

To determine whether TBAJ-876 is bacteriostatic or bactericidal against M. abscessus, we measured the survival of M. abscessus Bamboo upon drug exposure for 5 days at concentrations ranging from 3- to 300-fold the MIC. TBAJ-876 effectively suppressed bacterial growth at all concentrations tested but did not reduce the bacterial burden below the initial inoculum, similar to BDQ (Fig. 2), which was previously shown to be highly bacteriostatic against M. abscessus *in vitro* (36).

### TBAJ-876 does not antagonize the activity of commonly used anti-M. abscessus antibiotics.

Since treatment of M. abscessus infections requires drug combinations, we investigated pharmacodynamic interactions of TBAJ-876 with standard-of-care anti-M. abscessus antibiotics. We measured the growth inhibition activity of TBAJ-876 combined with clarithromycin, amikacin, cefoxitin, or imipenem against M. abscessus Bamboo, using the “checkerboard titration” assay. TBAJ-876 was also combined with rifabutin, which was recently shown to be active against M. abscessus and a potential repurposing candidate (52, 55–58). The fractional inhibitory concentration index (FICI) was calculated to characterize the interaction between TBAJ-876 and each of the test drugs (59). All TBAJ-876 combinations were largely additive, similar to BDQ observations (Table 3). Thus, TBAJ-876 does not exert any observable antagonism with major antibiotic classes used to treat M. abscessus disease.

**TABLE 3:** *In vitro* interaction of TBAJ-876 with selected drugs against M. abscessus Bamboo^*a*^

Drug A (class)	Combination with DARQ (drug B)	MIC (μM) of:				FICI^*b*^	Outcome^*b*^
		Drug A alone	Drug A in combination	Drug B alone	Drug B in combination		
Clarithromycin (macrolide)	TBAJ-876	0.53	0.24	0.46	0.13	0.74	Additivity
	BDQ		0.13	0.55	0.22	0.65	Additivity

Amikacin (aminoglycoside)	TBAJ-876	44	9.6	0.46	0.09	0.41	Synergy
	BDQ		14.7	0.55	0.07	0.46	Synergy

Cefoxitin (β-lactam)	TBAJ-876	43	9.7	0.46	0.09	0.42	Synergy
	BDQ		12	0.55	0.11	0.48	Synergy

Imipenem (β-lactam)	TBAJ-876	360	32	0.46	0.21	0.55	Additivity
	BDQ		56	0.55	0.21	0.54	Additivity

Rifabutin (rifamycin)	TBAJ-876	4.2	2.4	0.46	0.05	0.68	Additivity
	BDQ		1.0	0.55	0.20	0.60	Additivity

a The experiment was carried out three times independently, and the MICs are displayed as mean values. BDQ was used as a positive control.

b The FICI was calculated as (MIC of drug A in combination/MIC of drug A alone) + (MIC of DARQ B in combination/MIC of DARQ B alone) (71). A FICI of ≤0.5 indicates synergy, a FICI of >0.5 to 4 indicates additivity (no interaction), and a FICI of >4 indicates antagonism (59).

### TBAJ-876 is active in a mouse model of M. abscessus infection.

**FIG 3:**
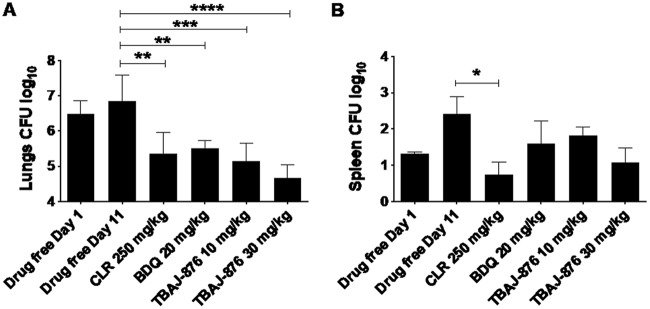
*In vivo* activity of TBAJ-876 against M. abscessus in NOD SCID mice. Shown are the effects of TBAJ-876 and the positive controls, clarithromycin (CLR) and BDQ, on bacterial loads in lungs (A) and spleen (B) of infected mice. M. abscessus subsp. *abscessus* K21 was used for the infection studies, as described previously (58). Mice, which were intranasally infected with ∼10^6^ CFU for 1 day, underwent drug treatment for 10 consecutive days. TBAJ-876 (10 or 30 mg/kg), clarithromycin (250 mg/kg), and BDQ (20 mg/kg) were administered once daily by oral gavage to groups of 6 mice per study arm. At 11 days postinfection, organ homogenates were plated on agar to determine the bacterial load. Results were analyzed using one-way analysis of variance (ANOVA) multicomparison and Tukey’s posttest (*, *P < *0.05; **, *P < *0.01; ***, *P < *0.001; ****, *P < *0.0001). The MICs of TBAJ-876 and BDQ against the strain were 0.09 μM and 0.3 μM, respectively. The experiment was carried out twice, and one representative data set is shown. The results are represented as mean values, with the standard deviations displayed as error bars.

To determine whether TBAJ-876’s attractive *in vitro* potency against M. abscessus translates into *in vivo* efficacy, NOD SCID mice were infected intranasally (58) and received daily oral doses of 10 and 30 mg/kg of body weight (the projected human efficacious doses) of TBAJ-876, starting at 1 day postinfection and for 10 consecutive days. Drug efficacy was defined as a statistically significant reduction of CFU in a study group relative to the vehicle control at the end of the experiment. The macrolide clarithromycin and BDQ, serving as positive controls at human-equivalent doses, significantly reduced lung bacterial loads by approximately 1 log CFU (Fig. 3A). At 10 mg/kg, TBAJ-876 was as efficacious as BDQ. Increasing the dose of TBAJ-876 to 30 mg/kg resulted in increased efficacy in both lungs and spleen, although the difference was not statistically significant (Fig. 3A and B). Thus, TBAJ-876 reduces lung and spleen M. abscessus burdens *in vivo* with an efficacy similar to that of BDQ.

## DISCUSSION

TBAJ-876 is a 3,5-dialkoxypyridine BDQ analogue discovered through a recent medicinal chemistry campaign (45) and currently developed against tuberculosis. Compared to BDQ, this new compound displays low hERG channel inhibition and less pronounced accumulation in tissues while retaining similar efficacy in mice infected with M. tuberculosis (45). Hence, TBAJ-876 presents itself as a potential next-generation BDQ with improved pharmacokinetic and tolerability profiles for the treatment of tuberculosis. Since BDQ is active against M. abscessus, we determined whether this holds true for TBAJ-876. Here, we show that TBAJ-876 and BDQ are similarly active against M. abscessus *in vitro*. TBAJ-876 exhibits similar potencies against reference strains of the M. abscessus complex and against a collection of clinical isolates representing the three subspecies of M. abscessus. Importantly, TBAJ-876’s *in vitro* activity translates into *in vivo* efficacy, as demonstrated in a mouse model of M. abscessus infection. Hence, we provide convincing preclinical evidence that TBAJ-876 may be useful not only for the treatment of tuberculosis but also for the treatment of lung disease caused by M. abscessus.

Furthermore, we show that TBAJ-876, similar to BDQ (60), does not antagonize the activity of drugs commonly used for the treatment of M. abscessus infection and could thus be safely coadministered with the standard of care, provided that there is an absence of pharmacokinetic drug-drug interactions. In the treatment of tuberculosis, BDQ is not administered concomitantly with rifampin because it is a substrate of CYP3A4, induced by rifampin, thus causing drug-drug interactions (61). Whether TBAJ-876 is metabolized by CYP3A4 in patients remains to be determined. Regardless, rifabutin has a much reduced CYP3A4 induction activity compared to rifampin (62), making it the rifamycin of choice in patient populations receiving antiretroviral therapies that include CYP3A4 substrates (63).

Given its ability to inhibit energy metabolism via inhibition of the mycobacterial F-ATP synthase (47), TBAJ-876 could also potentiate the activity of several other antibiotics through the depletion of intrabacterial ATP, which in turn would affect the activity of ATP-binding cassette (ABC) transporters (64, 65) acting as membrane-bound efflux pumps (66). Inhibition of these pumps via ATP starvation could impede drug efflux, which has emerged as an important determinant of drug resistance in M. abscessus (28).

While we show that TBAJ-876 does not antagonize growth inhibition by imipenem or cefoxitin, a recent study suggests antagonism between BDQ and β-lactams when the readout is bactericidal activity (67). BDQ was shown to block a lethal ATP burst induced by imipenem and cefoxitin, thus eliminating the bactericidal activity of these cell wall synthesis inhibitors against M. abscessus (67). Hence, caution may have to be exercised regarding the coadministration of TBAJ-876 with β-lactams in the clinical setting.

In conclusion, this study has shown that the new BDQ analogue TBAJ-876 is active *in vitro* and *in vivo* against the M. abscessus complex. This compound represents a potential alternative to BDQ, with improved pharmacokinetic and tolerability profiles, for the treatment of M. abscessus lung disease.

## MATERIALS AND METHODS

### Bacterial strains, culture media, and chemicals.

The reference strains of the subspecies of the M. abscessus complex were purchased from the American Type Culture Collection (ATCC) and Culture Collection University of Goteborg (CCUG). M. abscessus subsp. *abscessus* ATCC 19977 and M. abscessus subsp. *bolletii* CCUG 50184-T harbor the inducible clarithromycin resistance-conferring *erm*(41) T28 sequevar (65, 68). M. abscessus subsp. *massiliense* CCUG 48898-T harbors the nonfunctional *erm*(41) deletion sequevar, and hence, it is susceptible to clarithromycin (69).

M. abscessus Bamboo is a clinical isolate from a patient with amyotrophic lateral sclerosis and bronchiectasis. The strain was provided by Wei Chang Huang (Taichung Veterans General Hospital, Taichung, Taiwan). This strain was previously whole-genome sequenced, which showed that it belongs to M. abscessus subsp. *abscessus* and harbors the inactive, clarithromycin-sensitive *erm*(41) C28 sequevar (GenBank accession no. MVDX00000000) (51).

The other eight M. abscessus clinical isolates (M9, M111, M199, M232, M337, M421, M422, and M506), used for *in vitro* characterization of TBAJ-876’s activity, were provided by Jeanette W. P. Teo (Department of Laboratory Medicine, National University Hospital, Singapore). The subspecies and *erm*(41) sequevars of these isolates were determined previously (52).

For the *in vivo* efficacy study, the clinical isolate M. abscessus subsp. *abscessus* K21 was used. This strain harbors the inactive, clarithromycin-sensitive *erm*(41) C28 sequevar as determined previously (58). M. abscessus K21 was isolated from a patient and provided by Won-Jung Koh (Division of Pulmonary and Critical Care Medicine, Samsung Medical Center, Seoul, South Korea).

All M. abscessus strains were maintained in Middlebrook 7H9 medium (BD Difco) supplemented with 0.2% (vol/vol) glycerol (Fisher Scientific), 0.05% (vol/vol) Tween 80 (Sigma-Aldrich), and 10% (vol/vol) Middlebrook albumin-dextrose-catalase (ADC) (BD Difco). Cation-adjusted Mueller-Hinton (CAMH) broth (BD Difco) was also used to culture M. abscessus strains and was prepared according to the manufacturer’s instructions.

TBAJ-876 was synthesized by Bioduro LLC (Beijing, China) as described in the supplemental material, while BDQ was purchased from MedChem Express. Both TBAJ-876 and BDQ were dissolved in 100% dimethyl sulfoxide (DMSO) (MP Biomedicals) and sterilized using 0.2-μm polytetrafluoroethylene (PTFE) membrane filters (Acrodisc; Pall) before use.

### Growth inhibition dose-response assay.

The broth microdilution method (70) was utilized for carrying out a growth inhibition dose-response assay. Briefly, each well of clear 96-well flat-bottom Costar cell culture plates (Corning) was filled with 100 μl of liquid medium (complete 7H9 medium or CAMH broth). TBAJ-876 or BDQ was added to the first well in each row of the plate to create two times the desired highest final concentration. A 10-point 2-fold serial dilution of TBAJ-876/BDQ was carried out starting from this first well. The M. abscessus strains used for this assay were grown to mid-exponential phase and then diluted to an optical density at 600 nm (OD_600_) value of 0.1 using the same liquid medium used to run the assay. One hundred microliters of the diluted culture was added to each well to create a final OD_600_ value of 0.05 in each well. The plates were incubated for 3 days at 37°C, with shaking at 110 rpm using an orbital shaker. After the incubation period, the cultures in all wells were manually resuspended, and the OD_600_ of each well was read using a Tecan Infinite Pro 200 plate reader. The reported MIC values represent the concentration that inhibits 90% of bacterial growth compared to the untreated culture.

### Measurement of growth and survival of M. abscessus Bamboo under TBAJ-876 or BDQ treatment.

M. abscessus Bamboo was cultured in complete 7H9 medium and grown to mid-exponential phase. Subsequently, the culture was diluted to ∼10^6^ CFU/ml using complete 7H9 medium. Two milliliters of the diluted culture was transferred to each 14-ml round-bottom tube (SPL Life Sciences). TBAJ-876 and BDQ were added to their respective tubes to achieve final concentrations of 3-, 30-, or 300-fold their MICs. The tubes were incubated at 37°C under shaking at 160 rpm for 5 days. At the indicated time points, 10 μl of each sample was taken and diluted. For drug-free samples, 10 μl of 10^−3^ to 10^−7^ dilutions of each sample was plated out on Middlebrook 7H10 agar (BD Difco) supplemented with 0.2% (vol/vol) glycerol and 10% (vol/vol) Middlebrook oleic acid-albumin-dextrose-catalase (OADC) (BD Difco). For TBAJ-876- or BDQ-treated samples, 10 μl of 10^−3^ to 10^−5^ dilutions of each sample was plated out. The agar plates were incubated for 4 days at 37°C, and subsequently, CFU were determined by counting the colonies. Graphs were generated using GraphPad Prism 5 software.

### Checkerboard titration assay for assessing *in vitro* interaction of TBAJ-876 or BDQ with other drugs.

A checkerboard titration assay was carried out as described previously (71, 72). Briefly, TBAJ-876 or BDQ and one drug (clarithromycin, amikacin, cefoxitin, imipenem, or rifabutin) were added to complete 7H9 medium-containing 96-well flat-bottom Costar cell culture plates. Twofold serial dilutions were done to allow 7 different concentrations of TBAJ-876 or BDQ (0 μM to 2 μM) to be tested for interaction with 10 different concentrations of the drugs (0 μM to 4 μM for clarithromycin, 0 μM to 100 μM for amikacin, 0 μM to 90 μM for cefoxitin, 0 μM to 400 μM for imipenem, and 0 μM to 80 μM for rifabutin). Hence, a total of 70 different concentration combinations were tested for each combination between TBAJ-876 or BDQ and a drug. Each 96-well plate had a 7H9 medium-only control well and a drug-free bacterial culture control well. M. abscessus Bamboo was cultured in complete 7H9 medium and grown to mid-exponential phase. Subsequently, the culture was diluted to an OD_600_ of 0.1 using complete 7H9 medium and added to each well in the 96-well plate to create a final OD_600_ value of 0.05. The plates were incubated for 3 days at 37°C, under shaking at 110 rpm on an orbital shaker. After the incubation period, the culture in each 96-well plate was manually resuspended, and the OD_600_ of each well was read using a Tecan Infinite Pro 200 plate reader. Calculation of the fractional inhibitory concentration index (FICI) was done to analyze the results. The FICI is calculated as (MIC of drug A in combination/MIC of drug A alone) + (MIC of DARQ B in combination/MIC of DARQ B alone) (71). This calculation was done only for wells which showed 90% inhibition of bacterial culture growth compared to drug-free bacterial culture wells. A FICI of ≤0.5 indicates synergy, a FICI of >0.5 to 4 indicates additivity (no interaction), and a FICI of >4 indicates antagonism (59).

### Determination of *in vivo* activity of TBAJ-876.

*In vivo* efficacy determinations were carried out as described previously, using 8-week-old female NOD.CB17-*Prkdc^scid^*/NCrCrl (NOD SCID) mice (Charles River Laboratories) and the M. abscessus subsp. *abscessus* K21 strain (58). Briefly, anesthetized animals were infected by intranasal delivery of ∼10^6^ CFU of M. abscessus subsp. *abscessus* K21. Acute infection was achieved within 1 day. Drugs or the vehicle control was administered once daily for 10 consecutive days by oral gavage to the mice, starting from 1 day postinfection. Clarithromycin was formulated in 0.5% carboxymethyl cellulose–0.5% Tween 80–sterile water and administered at a dose of 250 mg/kg. TBAJ-876 and BDQ (as the free-base form) were formulated in 20% (2-hydroxypropyl)-β-cyclodextrin and administered at doses of 10 mg/kg and 30 mg/kg (TBAJ-876) or 20 mg/kg (BDQ). The BDQ dose of 20 mg/kg was selected to achieve efficacious exposure (area under the concentration-time curve [AUC]/MIC) comparable to that of 30 mg/kg TBAJ-876, based on the previously reported pharmacokinetic properties of both antibiotics (45). All mice were euthanized 24 h after the last dose (11 days postinfection), and their lungs and spleen were aseptically removed prior to homogenization. The bacterial load in these organs was determined by plating serial dilutions of the organ homogenates onto Middlebrook 7H11 agar (BD Difco) supplemented with 0.2% (vol/vol) glycerol and 10% (vol/vol) OADC. The agar plates were incubated for 5 days at 37°C prior to counting of colonies. All experiments involving live animals were approved by the Center for Discovery and Innovation Institutional Animal Care and Use Committee.

## Supplementary Material

Supplemental file 1
