# Supplementary material for: TBAJ-876, a 3,5-Dialkoxypyridine Analogue of Bedaquiline, Is Active against Mycobacterium abscessus
Source: Antimicrob Agents Chemother. 2020 Mar 24;64(4):e02404-19. doi: 10.1128/AAC.02404-19 (PMC7179298; doi:10.1128/AAC.02404-19)
Supplement: Supplemental file 1 [file AAC.02404-19-s0001.pdf]

Synthesis of TBAJ-876

Synthesis and analytics were carried out by Bioduro LLC (Beijing). All solvents were of analytical grade and used without further purification unless specifically noted. Column chromatography was performed using silica gel (100–200 mesh) as stationary phase. ^{1}H NMR spectra were measured on a Bruker AVANCEIII 400 NMR spectrometer in $CDCl_3$ and DMSO-d_6. Mass spectra were measured using an Agilent 1200-6110 LC-MS apparatus. The four isomers of TBAJ-876 were separated by Chiral column (OJ-H column, 20×250 mm;) using a Waters Thar Supercritical fluid chromatography (SFC) 80 system. Mobile phases were gradients of 30% IPA/70% CO_2. Purity of the four isomers were >95%.

Scheme 1: 6-Bromo-2-methoxy-3-((2,3,6-trimethoxypyridin-4-yl)methyl)quinoline (Unit A). A 3-L, three-necked round-bottomed flask, containing a magnetic stirring bar, a low-temperature thermometer and an equalizing dropping funnel, was connected to a nitrogen flow line and charged with a solution of n-BuLi (720 ml, 1.8 mol, 2.5M) in THF. The flask was charged with a solution of 3-bromo-2,6-dimethoxypyridine (250 g, 1.15 mol) in THF (800 ml), then cooled to -70 °C in a CryoCool bath (DLSB-5/40, ZhengZhouHengYan Instrument Co.). When the solution in the flask was at -70 °C, n-BuLi was added dropwise over 30 min. The reaction mixture was stirred at -70 °C for 45 min and subsequently, trimethoxyborane (154 g, 1.5 mol) was added dropwise over 10 min. The reaction mixture was stirred at -70 °C for 2 hrs and then heated to -10 °C. NaOH (574 ml, 1.72 mol) and H_2O_2 (324 g, 2.8 mol, 30%) were added dropwise to the mixture. The reaction mixture was stirred at 25 °C for 30 min and then stirred for a further 1 hr at 50 °C. Monitoring of the reaction mixture was carried out by LC-MS. After completion, saturated $Na_2S_2O_3$ (200 ml) aqueous solution was added to the mixture. Subsequently, the white solid 2,6-dimethoxypyridin-3-ol (A1) (101 g, 54%.) was obtained from the reaction mixture through extraction with EtOAc (3x500 ml), drying with Na_2SO_4,

filtration and evaporation. ^{1}H NMR (400 MHz, $CDCl_3$) δ 7.12 (d, J = 8.3 Hz, 1H), 6.21 (d, J = 8.2 Hz, 1H), 4.92 (s, 1H), 3.99 (s, 3H), 3.86 (s, 3H). Found: [M+H] = 156.1.

A solution of 2,6-dimethoxypyridin-3-ol (A1) (101 g, 647 mmol) in THF (500 ml) was stirred at 0 °C. NaH (53 g, 1.32 mol) was added in five portions to the mixture. Iodomethane (143 g, 1.01 mol) was then added and the mixture stirred at 25 °C for 12 hrs. After completion, the mixture was partitioned between EtOAc (400 ml) and water (400 ml). The organic layer was washed with water, dried with Na_2SO_4 and evaporated. The crude product was chromatographed on silica gel (1:1) to give 2,3,6-trimethoxypyridine (A2) (89 g, 81%) as a yellow oil. ^{1}H NMR (400 MHz, $CDCl_3$) δ 7.11 (d, J = 8.3 Hz, 1H), 6.22 (d, J = 8.3 Hz, 1H), 4.00 (s, 3H), 3.87 (s, 3H), 3.82 (s, 3H). Found: [M+H] = 170.0.

n-BuLi (303 ml, 758 mmol) was slowly added at -78 °C to a solution of 2,3,6-trimethoxypyridine (A2) (89 g, 523 mmol) in THF (600 ml). The mixture was stirred for 30 min. DMF (76.9 g, 1.05 mol) was then added and the mixture was stirred at -78 °C for 2 hrs. After completion, the mixture was partitioned between EtOAc (300 ml) and water (400 ml). The organic layer was washed with water, dried with Na_2SO_4 (20 g) and evaporated. The crude product was chromatographed on silica gel (PE / EtOAc = 1:1) to give 2,3,6-trimethoxyisonicotinaldehyde (A3) (34 g, 33%) as a yellow solid. ^{1}H NMR (400 MHz, DMSO-d_6) δ 10.28 (s, 1H), 6.47 (s, 1H), 3.98 (s, 3H), 3.86 (d, J = 2.8 Hz, 6H). Found: [M+H] = 197.9.

n-BuLi (81.6 mL, 204 mmol) was added dropwise, at -50 °C under N_2, to a solution of 2,2,6,6-tetramethylpiperidine (29.2 g, 207 mmol) in freshly distilled THF (400 mL). The mixture was stirred at -40 ℃ for 30 min and then cooled to -78 °C. A solution of 6-bromo-2-methoxyquinoline (45 g, 189 mmol) in dry THF (150 mL) was added dropwise to the mixture at -78 °C. The mixture was stirred at the same temperature for 1.5 hrs. A solution of 2,3,6-trimethoxyisonicotinaldehyde (A3) (34 g, 172 mmol) in dry THF (150 mL) was added dropwise at -78 °C to the reaction mixture, which was then stirred at -78 °C for 2.5 hrs. After

completion, water was added, and the aqueous mixture was extracted with EtOAc (3x200 ml). The organic phase was washed with saturated aqueous NaCl, dried with Na_2SO_4 (15 g) and concentrated to give a yellow residue. This residue was purified by flash column chromatography using PE: EtOAc (3:1) to give (6-bromo-2-methoxyquinolin-3-yl)(2,3,6-trimethoxypyridin-4-yl)methanol (A4) (23.2 g, 31%) as a white solid. [M+H] =435.1.

NaH (3.16 g,79 mmol) was added, at 0 °C under N_2, to a solution of (6-bromo-2-methoxyquinolin-3-yl)(2,3,6-trimethoxypyridin-4-yl)methanol (A4) (23 g,53 mmol) in THF (200 mL). The mixture which was stirred at 25 °C for 1 hr. CS_2 (5.8 g,76 mmol) was then added dropwise at 0 °C. The reaction mixture was stirred at 25 °C for 1 hr and then iodomethane (11.35 g, 80 mmol) was added dropwise at 0 °C. The mixture was stirred at 25 °C for 15 min and then 3 drops of water was added. After 20 min, the reaction was quenched with ice-water (200 g) and extraction was carried out using EtOAc (300 ml). The organic phase was washed with saturated aqueous NaCl, dried with Na_2SO_4 (10 g) and concentrated to give a yellow residue. Purification by flash column chromatography using DCM: PE (1:2) gave O-((6-bromo-2-methoxyquinolin-3-yl)(2,3,6-trimethoxypyridin-4-yl)methyl)S-methylcarbonodithioate (A5) (23.5 g, 84%) as a white solid. [M+H] = 525.1.

Tributylstannane (15.6 g, 54 mmol) under N_2 was added to a solution of O-((6-bromo-2-methoxyquinolin-3-yl)(2,3,6-trimethoxypyridin-4-yl)methyl)S-methylcarbonodithioate (A5) (23.5 g, 45 mmol) in 200 mL toluene. The mixture was heated to 80 °C and then azodiisobutyronitrile (1.84 g, 11 mmol) was added. The mixture was heated to 100 °C for 2 hrs. After completion, the mixture was stirred at room temperature and then water was added. The aqueous mixture was extracted with EtAOc (3x200 ml). The organic phase was washed with saturated aqueous NaCl, dried over Na_2SO_4 and concentrated to give a yellow residue. Purification by flash column chromatography using elute PE: EtOAc (1:1) gave 6-bromo-2-methoxy-3-((2,3,6-trimethoxypyridin-4-yl)methyl)quinoline (Unit A) (10.8 g, 58%) as a white

solid. ^{1}H NMR (400 MHz, DMSO-d_6) δ 8.11 (d, J = 1.5 Hz, 1H), 7.85 (s, 1H), 7.73 – 7.66 (m, 2H), 6.09 (s, 1H), 4.00 (s, 3H), 3.95 (s, 2H), 3.92 (s, 3H), 3.78 (s, 3H), 3.62 (s, 3H). Found: [M+H] = 418.7

Scheme 2: 1-(2,6-Dimethoxypyridin-4-yl)-3-(dimethylamino)propan-1-one (Unit B). A solution of 2,6-dichloroisonicotinic acid (50 g, 262 mmol) in CH_3OH (500 ml) was stirred at 0 °C. NaH (26.2 g, 655 mmol) was added in five portions to the mixture, which was subsequently stirred at 60 °C for 5 hrs. Water (400 ml) was added to the solution and pH was adjusted to 3 using 2M HCl solution. Extraction with EtOAc (3x400ml), drying with Na_2SO_4 (10 g) and filtration gave a solvent that was evaporated to give 2-chloro-6-methoxyisonicotinic acid (B1) (38 g, 78%) as a white solid. Found: [M+H] = 188.1.

A solution of 2-chloro-6-methoxyisonicotinic acid (B1) (38 g, 202 mmol) in DMF (400 ml) was stirred at 0 °C. NaH (20 g, 500 mol) was added in five portions to the mixture, which was subsequently stirred at 150 °C for 5 hrs. Water (400 ml) was added to the solution and pH was adjusted to 3 using 2M HCl solution. Extraction with EtOAc (3x400ml), drying with Na_2SO_4 (10 g) and filtration gave a solvent that was evaporated to give 2,6-dimethoxyisonicotinic acid (B2) (32.7 g, 88%) as a white solid. Found: [M+H] = 184.1.

A round-bottom flask was charged with 2,6-dimethoxyisonicotinic acid (B2) (32.7 g, 178 mmol), HBTU (81 g, 214 mmol), Et_3N (54 g, 535 mmol) and N,O-dimethylhydroxylamine (13.05 g, 214 mmol) in dichloromethane (1 L). The mixture was stirred at 20 °C for 12 hrs. The reaction was detected by LC-MS. Subsequently, water (500 ml) was added to the resultant solution. Extraction with EtOAc (3x500 ml), drying with Na_2SO_4 and filtration gave a solvent, which was evaporated to give N,2,6-trimethoxy-N-methylisonicotinamide (B3) (31.1 g, 77%) as a yellow solid. ^{1}H NMR (400 MHz, DMSO-d_6) δ 6.46 (s, 2H), 3.89 (d, J = 5.1 Hz, 6H), 3.57 (s, 3H), 3.24 (s, 3H). Found: [M+H] =227.0.

Vinyl magnesium bromide (411 ml, 411 mmol) in THF (300 ml) was slowly added at -78 °C to a solution of N,2,6-trimethoxy-N-methylisonicotinamide (B3) (31.1 g, 137 mmol) in THF. The mixture was stirred for 30 min and dimethylamine (685 ml, 685 mmol) was then added in quick succession. The mixture was stirred at -60 °C for 2 hrs and subsequently extracted with EtOAc (3×100 mL). The combined extraction was washed with water and brine, dried over sodium sulfate and concentrated under vacuum to give 1-(2,6-dimethoxypyridin-4-yl)-3-(dimethylamino)propan-1-one (Unit B) (15 g, 47 %). ^{1}H NMR (400 MHz, DMSO-d_6) δ 6.74 (s, 2H), 3.88 (s, 6H), 3.08 (t, J = 7.0 Hz, 2H), 2.55 (t, J = 7.0 Hz, 2H), 2.13 (s, 6H). Found: [M+H] = 239.0.

Scheme 3: 1-(6-Bromo-2-methoxyquinolin-3-yl)-2-(2,6-dimethoxypyridin-4-yl)-4-(dimethylamino)-1-(2,3,6-trimethoxypyridin-4-yl)butan-2-ol (TBAJ-876). n-Butyllithium (13 ml, 32.5 mmol) was added at -50 °C under N_2 to a solution of 2,2,6,6-tetramethylpiperidine (4.86 g, 34 mmol) in THF (100 mL). The reaction mixture was stirred at -40 °C for 20 min and then stirred for a further 30 min at 25 °C. A solution of 6-bromo-2-methoxy-3-((2,3,6-trimethoxypyridin-4-yl)methyl)quinoline (Unit A) (10.8 g, 26 mmol) in THF (50 ml) was added dropwise to the mixture at -50 °C. The mixture was stirred at the same temperature for 2 hrs. Subsequently, a solution of 1-(2,6-dimethoxypyridin-4-yl)-3-(dimethylamino)propan-1-one (Unit B) (7.02 g, 29 mmol) in THF (500 mL) was added dropwise to the mixture at -60 °C. The mixture was stirred at the same temperature for 2 hrs. After completion, saturated aqueous NH_4Cl (50 ml) was added. The aqueous mixture was extracted with EtOAc (3x100 ml). The organic phase was washed with saturated aqueous NaCl, dried with Na_2SO_4 (10 g) and concentrated to give a yellow residue. Purification by flash column chromatography using PE: EA (1:1) gave 1-(6-bromo-2-methoxyquinolin-3-yl)-2-(2,6-dimethoxypyridin-4-yl)-4-(dimethylamino)-1-(2,3,6-trimethoxypyridin-4-yl)butan-2-ol (4 isomers of TBAJ-876) (3.5 g, 21%). The four isomers of TBAJ-876 (P1 – P4) were separated by Chiral column (OJ-

H column, 20×250 mm;) using a Waters Thar SFC 80 system. Mobile phases were gradients of 30% IPA/70% CO_2 at 40 g/min. Found: [M+H] = 658.6.

TBAJ-876 (P1): ^{1}H NMR (400 MHz, $CDCl_3$) δ 8.18 (s, 1H), 7.86 (d, J = 2.1 Hz, 1H), 7.70 (d, J = 8.9 Hz, 1H), 7.63 (dd, J = 8.9, 2.2 Hz, 1H), 7.23 (s, 1H), 6.58 (s, 1H), 5.52 (s, 1H), 4.24 (s, 3H), 3.90 (s, 6H), 3.83 (d, J = 1.8 Hz, 6H), 3.43 (s, 3H), 2.38 – 2.19 (m, 1H), 2.13-1.54 (s, 1H).

TBAJ-876 (P2): ^{1}H NMR (400 MHz, $CDCl_3$) δ 8.18 (s, 1H), 7.86 (d, J = 2.1 Hz, 1H), 7.70 (d, J = 8.9 Hz, 1H), 7.63 (dd, J = 8.9, 2.2 Hz, 1H), 7.23 (s, 1H), 6.58 (s, 1H), 5.52 (s, 1H), 4.24 (s, 3H), 3.90 (s, 6H), 3.83 (d, J = 1.8 Hz, 6H), 3.43 (s, 3H), 2.38 – 2.19 (m, 1H), 2.13-1.54 (s, 11H).

TBAJ-876 (P3): ^{1}H NMR (400 MHz, $CDCl_3$) δ 8.75 (s, 1H), 7.84 (d, J = 1.9 Hz, 1H), 7.59 – 7.50 (m, 2H), 6.57 (s, 2H), 5.42 (s, 1H), 4.03 (s, 3H), 3.96 (s, 3H), 3.90 (s, 3H), 3.83 (d, J = 4.3 Hz, 9H), 2.27 (t, J = 12.6 Hz, 1H), 2.17 – 1.82 (m, 9H), 1.80 – 1.68 (m, 1H), 1.60 (s, 1H).

TBAJ-876 (P4): ^{1}H NMR (400 MHz, $CDCl_3$) δ 8.75 (s, 1H), 7.84 (d, J = 1.9 Hz, 1H), 7.59 – 7.50 (m, 2H), 6.57 (s, 2H), 5.42 (s, 1H), 4.03 (s, 3H), 3.96 (s, 3H), 3.90 (s, 3H), 3.83 (d, J = 4.3 Hz, 9H), 2.27 (t, J = 12.6 Hz, 1H), 2.17 – 1.82 (m, 9H), 1.80 – 1.68 (m, 1H), 1.60 (s, 1H).

The biologically active 1R/2S diastereomer of TBAJ-876 (1) is P1 and hence, P1 was used for characterisation in this study.

REFERENCES

1. Sutherland HS, Tong AS, Choi PJ, Blaser A, Conole D, Franzblau SG, Lotlikar MU, Cooper CB, Upton AM, Denny WA, Palmer BD. 2019. 3,5-Dialkoxypyridine analogues of bedaquiline are potent antituberculosis agents with minimal inhibition of the hERG channel. Bioorg Med Chem 27:1292-1307.
